# Predicting inpatient violence using an extended version of the Brøset-Violence-Checklist: instrument development and clinical application

**DOI:** 10.1186/1471-244X-6-17

**Published:** 2006-04-25

**Authors:** Christoph Abderhalden, Ian Needham, Theo Dassen, Ruud Halfens, Hans-Joachim Haug, Joachim Fischer

**Affiliations:** 1 University Bern Psychiatric Services, Nursing and Social Education Research Unit, Bolligenstrasse 111, 3000 Bern 60, Switzerland; 2 University of Applied Science, Route des Cliniques 15, 1700 Fribourg, Switzerland; 3 Humboldt-University, Department of Nursing Science, Schumannstr. 20/21, 10117 Berlin, Germany; 4 Universiteit Maastricht, Faculty of Health science, P.O. Box 616, 6200 MD Maastricht, The Netherlands; 5 University Zurich and, Psychiatric Hospital Schloessli, 8618 Oetwil am See, Switzerland; 6 Department of Public Health, Social and Preventive Medicine, Mannheim Medical Faculty, University of Heidelberg, Theodor Kutzer Ufer 1, D-68135 Mannheim, Germany

## Abstract

**Background:**

Patient aggression is a common problem in acute psychiatric wards and calls for preventive measures. The timely use of preventive measures presupposes a preceded risk assessment. The Norwegian Brøset-Violence-Checklist (BVC) is one of the few instruments suited for short-time prediction of violence of psychiatric inpatients in routine care. Aims of our study were to improve the accuracy of the short-term prediction of violence in acute inpatient settings by combining the Brøset-Violence-Checklist (BVC) with an overall subjective clinical risk-assessment and to test the application of the combined measure in daily practice.

**Method:**

We conducted a prospective cohort study with two samples of newly admitted psychiatric patients for instrument development (219 patients) and clinical application (300 patients). Risk of physical attacks was assessed by combining the 6-item BVC and a 6-point score derived from a Visual Analog Scale. Incidents were registered with the Staff Observation of Aggression Scale-Revised SOAS-R. Test accuracy was described as the area under the receiver operating characteristic curve (AUC_ROC_).

**Results:**

The AUC_ROC _of the new VAS-complemented BVC-version (BVC-VAS) was 0.95 in and 0.89 in the derivation and validation study respectively.

**Conclusion:**

The BVC-VAS is an easy to use and accurate instrument for systematic short-term prediction of violent attacks in acute psychiatric wards. The inclusion of the VAS-derived data did not change the accuracy of the original BVC.

## Background

Patient aggression is a common problem in acute psychiatric wards. Violent outbursts threaten the health, safety and well-being of other patients and staff. Psychiatric nurses are at particularly high risk of being victimized. However, psychiatric staff is not only a passive target of potential patient violence. Violence management is a key component of clinical practice, and psychiatric staff performs a wide range of interventions to modulate the context and the interaction with potentially violent patients. Preventive measures are of special importance. The timely use of preventive measures presupposes a preceded risk assessment. Therefore, accurate risk prediction to allow targeted interventions is of paramount importance [1].

Several attempts have been made to introduce accurate measures for risk prediction [2]. Generally spoken fall into two categories: actuarial methods and prediction models derived from acute patient observation [2-4]. Actuarial models predict risk from the presence of statistically derived risk factors like age, gender, psychopathological state, diagnosis etc. Most studies using this method found that patients who had exhibited violent behavior in the past were substantially more likely to become aggressive during a new hospitalization than those with no history of aggressive behavior [5,6]. The main criticisms advanced towards actuarial methods is a) that they discard the experience of the staff currently dealing with the patient, b) that they perform less well in non-forensic or acute settings [5,7] and c) that they require the collection of data that may not be readily available in acutely admitted patients [1,8].

Clinical prediction models based on acute patient observation use different approaches, considering factors as e.g. psychopathological states. One approach is based on overt patient behavior. A recently published method is the Brøset Violence Checklist (BVC), which has been validated in Norwegian and German [9-11]. The BVC assesses the presence of six observable patient behaviors namely whether the patient is confused, irritable, boisterous, verbally threatening, physically threatening, and attacking objects. The reported discriminatory ability is good with a correct prediction rate around 85% [10]. Another clinical model emphasizes the staff's ability to judge the risk by integrating all available information into a formal subjective risk prediction statement. This subjective prediction is operationalized by likert-type scales or Visual Analogue Scales [12-16]. Investigators applying this approach found correct prediction rates of 75% [14]. The limitation to either approach is a considerable residual risk of false positives.

### Aim of the study

The aim of the present study was to ascertain whether combining both methods would yield improved risk prediction over either method alone. The study comprised two independent patient samples from different hospitals. The first patient sample served as a derivation dataset to identify the optimal algorithm for combining the BVC and the subjective prediction. The second patient sample served as the validation dataset, in which the prediction method was applied to clinical practice.

## Methods

### Design

Two independent prospective cohort studies were conducted. The first served to develop the risk assessment instrument (derivation sample). The second patient sample tested the clinical application of the method (validation sample).

The study protocols were reviewed and approved by the research ethics boards of the Cantons Zurich (E-016/2001), Appenzell AR (10/01) and Berne (24.12.2001/IH/Hz/EW).

### Setting and sample

Both studies were conducted in acute psychiatric wards in the German speaking part of Switzerland. All participating wards were closed admission wards providing comprehensive psychiatric service to the respective catchment areas. The first sample (derivation dataset) consisted of 219 consecutively admitted patients to six wards within three hospitals during a two-moth period. The number of beds in each ward ranged from 15 to 19. The second sample (validation dataset) consisted of 300 consecutively admitted patients to two wards during a six-month period. These two 12 bed wards were situated in two different hospitals in different cantons (one rural area, one urban area) to assure independence from the derivation dataset.

### Instrument development

During instrument development psychiatric nurses responsible for the care of the patient provided an assessment during admission and twice daily (10 a.m. and 6 p.m.) at admission day and during the next three days or until discharge/transferral. Therefore, the maximum number of ratings per patients was 9 in the case of an admission time earlier than the regular rating at 11 a.m. Lower numbers of ratings resulted from missing items and when patients were discharged from the ward prior to the third day after hospitalization. Assessment forms contained the German research version of the BVC and a Visual Analogue Scale (VAS) of 10 cm length. Nurses were asked to indicate the presence or absence of the six behaviors constituting the BVC. In addition, nurses encoded their subjective perception of risk for a physical attack within the next 12 hours on the VAS. The endpoints of the VAS were marked as "no risk" and "very high risk". The data collection form was also used to gather information about any preventive measures taken since the last rating. No clues were provided about the interpretation of the BVC or the VAS. From these data, the final instrument (BVC-VAS) was developed as described in the statistical analysis section. The objective of this instrument to be developed was to integrate the findings from the BVC and the Visual Analogue Scale to a summary score. Crafting an instrument that would be compatible with routine use required graphic refinement of the BVC as well as a simple method to translate VAS-readings into scoring points. The latter was achieved by constructing a slide rule that resembled the VAS on the front side and provided the VAS score reading on the backside. The final instrument was pre-tested in a different ward before application in the validation study.

### Instrument validation

The new instrument (BVC-VAS) was integrated into clinical routine in two admission wards in two hospitals. To test the instrument during practical application, staff was aware about the interpretation of the obtained scores. Like in the derivation sample, nurses assessed the risk of newly admitted patients on the day of admission and the following three days twice daily.

### Outcome measurements

The main outcome measure was the occurrence of physical attacks on persons during the next shift following assessment. The severity of the aggressive event was recorded using the Staff Observation of Aggression Scale Revised (SOAS-R) [17-19]. Test accuracy was described as the area under the receiver operating characteristic curve [20]. A secondary outcome was the implementation of intense preventive measures such as seclusion or forced injection of psychotropic drugs. While this outcome may not be regarded as independent from the prediction, it allows the evaluation of false positive cases, i. e. to examine whether patients were unable to perpetrate violent attacks because of intense preventive measures. Thus, some of the false positive predictions may in fact be a consequence of effective prevention [13,21].

### Statistical analysis

The overall aim of the development of the BVC-VAS was to arrive at a simple number scoring system with presentation of risk as natural frequencies (e.g. 1 out of 10 patients with this score will attack). Such presentation of results is believed to provide a better framework to base actions than simple categorization as low or high risk. The statistical analysis consisted of two steps: First, an optimized prediction score was derived from the derivation dataset with the aim to provide four distinct risk strata: high, moderate risk, low risk and very low risk. Second, the application of the scoring system was tested under realistic conditions in a validation sample.

During derivation we employed independent logistic regression analyses with attack, aggression and coercive measures as the binary outcome variable. To account for possible non-linear relation between risk and individual BVC items, we performed additional analyses by entering each item as individual variable and by recoding numbers of BVC items into dummy variables. Second, we explored the relation between the VAS-distance measured in mm and the occurrence of physical attacks by independent logistic regression analyses. Within the constraints from the small dataset, these analyses did not suggest superiority of the single coding of symptoms over the simpler adding of symptoms. Next, several transformations of the raw VAS score (logarithmic, quadratic) were carried out, of which the logarithmic transformation yielded the highest discriminatory power. Because replacing the log-transformed VAS with the scoring points did not alter the predictive accuracy, we proceeded to adding the BVC and the VAS to a common summary score. We checked which combination of BVC scores and VAS scores would yield the best performing model, by testing different weights of the two scores. However, due to the small number of observed events, logistic regression analyses failed to ascertain with statistical significance whether non-balanced weighing would have yielded improved diagnostic performance over giving equal weights to the subjective assessment and the BVC. Therefore, we proceeded with equal weights. Thus, the final scale consisted of 12 score points, of which up to 6 were contributed from the BVC and up to 6 from a logarithmic transformation of the VAS. Finally, we calculated multilevel likelihood ratios for ranges of the revised BVC score, to be able to enumerate risk rather than expressing risk with ambiguous wordings. For practicability we chose four risk segments, corresponding to very low risk, low risk, moderate risk and high risk. In the validation dataset we elucidated the discriminatory performance of the total score and each subscore by independent analyses with the respective outcomes (attack/attack or intense preventive measures) as the outcome variables. To compare models, we used the area under the receiver operating characteristic curve (AUC-ROC). The AUC-ROC is determined from plotting sensitivity against 1-specificity for all possible cut-offs, in case of the combined BVC-AUC score for values ranging from 0 to 12. An area of 1 indicates a perfect prediction; an area of 0.5 is a chance result. Few clinical scores achieve AUCs ranging above 0.75, tests with an AUC of 0.95 are considered excellent [20]. Analyses were carried out in SPSS version 10 (SPSS inc, Chicago, Illinois) for obtaining confidence intervals for area under the receiver operating characteristic curves and in SAS (version 8.2, SAS institute, Cary, North Carolina) for model development.

## Results

### Patient sample

The derivation sample consisted of 219 patients; the validation sample of 300 patients. Patients in both samples were similar regarding gender, lengths of stay, the proportion of involuntary admission and the proportion of patients having a ICD-10 F1, F2 and F3 diagnosis. The validation sample comprised a lower proportion of patients having ICD-10 F4/F6 diagnoses (Neurotic, stress-related or somatoform disorder/personality disorder), and the mean age was lower (Table 1).

**Table 1:** Patient characteristics

	Derivation sample	Validation sample	
Number	219	300	
Male (%)	60.3 %	61.0 %	ns
Age (mean± SD)	39.8± 12.9	36.4± 13.3	p = 0.002*
ICD-10 F1 (Alcohol and drug use disorders)	23.9 %	22.8 %	ns
ICD-10 F2 (Schizophrenic or delusional disorder)	34.9 %	40.1 %	ns
ICD-10 F3 (Affective disorder)	14.8 %	16.5 %	ns
ICD-10 F4/6 (Neurotic, stress-related or somatoform disorder/personality disorder)	20.6 %	13.6 % %	p = 0.042**
ICD-10 (Other diagnoses)	5.8 %	7.0 %	ns
Involuntary admissions	58 %	64 %	ns
Median length of stay (days)	23	22	ns
			
Physical attacks	14	37	
Patients involved in ≥ 1 attack	10 (4.6 %)	27 (9.0 %)	ns
Severity of attacks (SOAS-R) (mean; range)	12.7 (5 – 18)	13.4 (4 – 20)	ns
Intense preventive measures	52	94	
Patients with ≥ 1≥ intense preventive measure	28 (12.8 %)	41 (13.7 %)	n.s.

* *Mann-Whitney U-Test*

** Pearson chi^2^

### Instrument development phase

During the derivation study we obtained 1203 ratings for each of the two measures for the 219 patients (5.5 ratings per patient). During the study period 14 physical attacks toward staff occurred involving 10 patients. The severity of the incidents on the 22-point SOAS-severity scale ranged from 5 to 18. Fifty-three of the ratings were followed by implementation of intense preventive measures (injection of drugs or seclusion). The sensitivity of the original BVC (cut-off > 2 points) was 64.3% (95%-CI 35.1–87.2), the specificity was 93.9% (95%-CI 92.4–95.2%), yielding a positive predictive value in this low-prevalence sample of 11.1% (95%-CI 6%–20%). The area under the receiver operating characteristic curve (AUC_ROC_) amounted to AUC_ROC _= 0.88 (95%-CI 0.76 – 0.99). In 1/3 of the cases with a false positive result intense preventive measures were implemented following the rating. The subjective VAS based risk assessment yielded a slightly higher discriminatory ability than the BVC-rating with an AUC_ROC _= 0.93 (95%-CI 0.88 to 0.98). As explained in the methods section, a non-linear transformation of the VAS-distances to scoring points provided the best fit of the model. The final scoring solution is presented in Table 2.

**Table 2:** Transformation of VAS-data into 6-point-scale

VAS (mm)	Score (Points)
0	0
1 – 5	1
6 – 10	2
11 – 20	3
21 – 40	4
41 – 80	5
81 – 100	6

**Figure 1:**
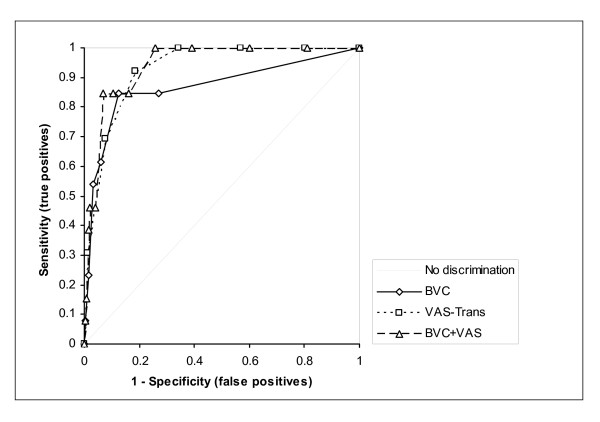
Receiver operating characteristic curves in the derivation dataset.

Adding these scores to the summed BVC resulted in a new scale ranging from 0 to 12. The test accuracy of the prediction for the equally weighted combined score (BVC-VAS) amounted to an AUC_ROC _of 0.94 (95%-CI 0.90 to 0.98) (Figure 1).

**Figure 2:**
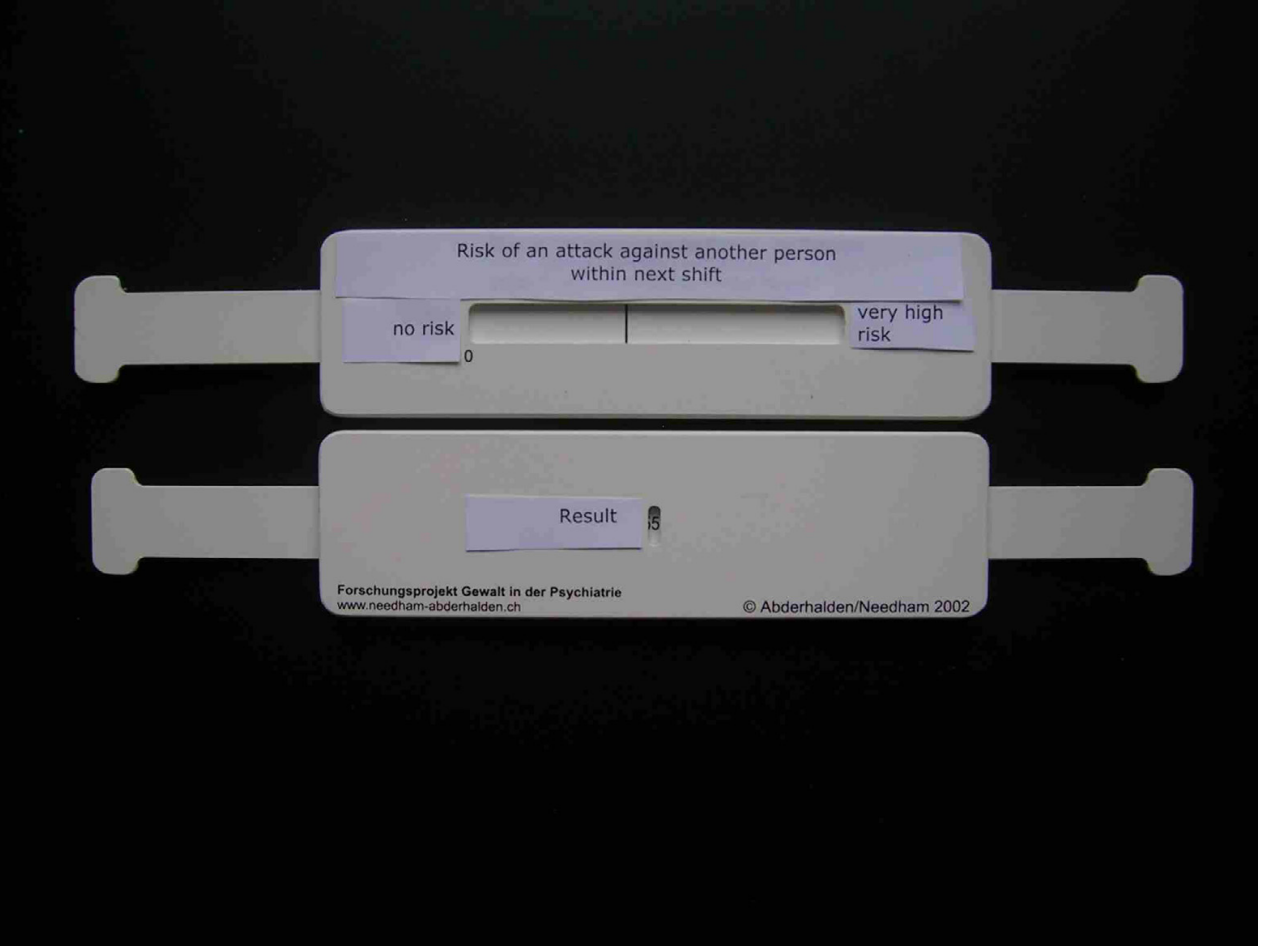
Slide rule.

For the clinical application study, we developed a slide rule style VAS to capture the subjective assessment of risk and to implement the logarithmic transformation of VAS distances into scores (see Figure 2). The front side shows the VAS scale, while the rear provides a window from which the log transformed score can be obtained. We also included a multilevel-likelihood based interpretation of the BVC-VAS score, in order to prevent the danger of overestimation of the potential aggressiveness of patients [22,23], aiming to avoid unjustified interventions. We specifically employed natural number frequency wordings instead of percentages to ease interpretation. Table 3 displays the wording of the resulting risk assessment.

**Table 3:** Interpretation of the extended version of the BVC, obtained from the derivation data-set

BVC-VAS Score	Odds ratio (95% CI) validation sample	Interpretation
0 – 3 Points	1.0 (reference group)	Very low risk (< 1 of 300 patients will attack a person)
4 – 6 Points	5.5 (2.0–15.0)	Low risk (about 1 out of 100 patients will attack a person)
7 – 9 Points	23.2 (8.7–62.1)	Moderate risk (about 1 out of 10 patients will attack a person)
10–12 Points	53.2 (16.9–168)	High risk (about 1 out of 4 to 5 patients risk will attack a person)

Due to the small number of attacks in the derivation dataset, modeling of risk categories as separate dummy variables was not possible. The odds ratio per increase in category (e.g. from 0-3 points to 4-6 points) amounted to 7.4 (95% CI 4.0-13.8). The interpretation is based on the observed incidence rate of attacks in a larger epidemiological study in Swiss acute psychiatry wards.

Because we were interested in testing the performance of the instrument in routine application we provided recommendations in addition to the risk enumeration. The scoring form suggested discussing the risk within the nursing team for patients scoring between 7 and 9 (moderate risk) and to consider the implementation of preventive measures from a list provided with the instrument. A score of 10 or more (high risk) constituted the obligation to discuss the risk AND to plan and implement preventive measures from the same list of possible preventive measures (see appendix).

### Validation phase

The validation phase comprised the clinical application of the BVS-VAS and involved 300 consecutive patients for whom 2084 ratings for each of the two measures were observed. Twenty seven percent of the patients had less than 6 ratings due to early discharge or transferral to another ward. During this period 37 attacks were registered, involving 27 patients. The instrument was well accepted by the nurses and was easily integrated into daily routine. The AUC_ROC _for the combined BVC-VAS amounted to 0.83 (95% CI: 0.75 – 0.90). While the original BVC alone showed a similar discriminatory performance as in the derivation dataset AUC_ROC _= 0.86 (95% CI: 0.79–0.92), the ruler-based visual analogue scale yielded a considerably lower accuracy with an AUC_ROC _= 0.74 (95% CI: 0.65–0.83). Because the staff was provided with an interpretation and recommendation of action in cases of moderate and high risk, we also determined the test accuracy for a composite endpoint combining the primary outcome (attack) and the secondary outcome (intense preventive measures). Using this event definition, the combined instrument yielded an AUC_ROC _= 0.90 (95% CI: 0.86–0.93), with a similar AUC_ROC _= 0.89 (95% CI: 0.86–0.92) for the original BVC, and a lower AUC_ROC _= 0.85 (95% CI: 0.81–0.89) for the ruler based VAS (see Tables 4 and 5).

**Table 4:** Summary of the areas under the receiver operating characteristic curves (95% Confidence interval)

Physical attack within next shift	Derivation sample	Validation sample
Original BVC	0.87 (0.74–0.99)	0.86 (0.80–0.93)
VAS (Transformed)	0.94 (0.89–0.97)	0.74 (0.65–0.83)
BVC-CH	0.95 (0.90–0.98)	0.83 (0.75–0.90)
		
**Physical attack OR intense preventive measure within next shift**	**Derivation sample**	**Validation sample**
Original BVC	0.82 (0.75–0.89)	0.88 (0.86–0.92)
VAS (Transformed)	0.87 (0.82–0.91)	0.85 (0.81–0.89)
BVC-CH	0.89 (0.84–0.94)	0.90 (0.86–0.93)

**Table 5:** Ratings and accuracy of predictions

	Derivation-Sample			Validation-Sample			Validation-Sample		
Outcome within the next shift	physical attack (n = 14)			physical attack (n = 37)			physical attack OR intense preventive measure ** (n = 121)		

Prediction method and cut-off points	BVC >= 3	BVC-VAS >= 7	VAS >= 4	BVC >= 3	BVC-VAS >= 7	VAS >= 4	BVC >= 3	BVC-VAS >= 7	VAS >= 4

Ratings	1203	1189*	1189	2084	2084	2084	2084	2084	2084
True Positives	9	11	12	25	20	21	74	82	91
False Negatives	5	2	1	12	17	16	47	39	30
True Negatives	1117	1095	959	1852	1884	1692	1817	1862	1678
False Positives	72	81	217	195	163	355	146	101	285
Sensitivity/Specificity in %	64/94	85/93	92/82	68/91	54/92	57/83	61/93	68/95	75/86

* *4 VAS-ratings missing*

**seclusion and/or mechanical restraint and/or forced injection

In the validation dataset, the Spearman's Rho correlation coefficient between the VAS and the BVC was r = 0.59, as compared to r = 0.50 in the derivation dataset.

## Discussion

The first aim of the study was to develop an extended version of the Brøset-Violence-Checklist that includes both the structured clinical assessment of observable patient behavior as well as the unaided subjective clinical assessment of psychiatric nurses on the patient's risk of perpetrating a violent attack. The second aim of the study was to test the instruments test accuracy and application in clinical practice. To this end, we conducted a prospective cohort study involving separate samples for instrument development (derivation sample) and clinical application (validation sample). The main findings of the study were that the visual analogue scale slightly improved the diagnostic accuracy in the derivation dataset (where no interpretation was provided), but that this effect was not retained in the validation dataset (where interpretation of the score was available). In the validation dataset the test accuracy of the VAS was significantly lower than in the derivation dataset. In contrast, the performance of the BVC was identical in both samples.

What are the clinical implications of these findings? The original BVC checklist proved to be remarkably stable in the independent dataset. Apparently, the BVC checklist combines the virtues of a structured clinical method by inquiring about specific patient behaviors. While it is still left to the discretion of the rater to decide, whether a specific behavior is actually present or not (e.g. being boisterous). Such subjective decisions may be more reliable than the subjective overall assessment provided in a Visual Analogue Scale. Moreover, we cannot rule out that providing the interpretation of the score affected the ratings. Of the two assessment methods, the BVC score is closer to resembling the practice of actuarial scores. The replication of almost identical test accuracy to the original Norwegian study in two independent samples underscores the possible generalizability of the instrument. Notwithstanding these encouraging findings, a relevant issue remains the limited positive predictive value in our settings with a low prevalence of physical attacks. This underscores the need for cautious interpretation of positive results and reporting of multilevel likelihood ratios. The satisfactory test accuracy (AUC_ROC _= 0.90) of the combined instrument when using the composite endpoint emphasizes the applicability in daily routine. Our data do not support the presumption that the test accuracy improved to a relevant extent by including the subjective element of the visual analogue scale. We hesitate to recommend to solely using the VAS, for three reasons: First, in the derivation dataset nurses were unaware of the interpretation of the VAS rating and its clinical implication. A significantly lower test accuracy of the VAS was observed in the validation dataset, were scoring mattered – suggesting possible assessment biases. Second, a checklist of observable behaviour is not only helpful for less experienced staff, but also facilitates communication. Third, the VAS-results has to be regarded as product of the hidden process of clinical reasoning (black-box). However, the nurses' feedback on the user friendliness of the combined instrument as compared to our previous experience when using the BVC alone suggested an increased compliance and acceptance of the instrument. Therefore, we have opted for using the combined instrument in the ongoing randomized controlled trial evaluating the efficacy of systematic prediction on occurrence rates of violent attacks and intense coercive measures.

Several caveats of the study must be acknowledged. A purist approach to the validation study would have mandated employing exactly the same presentation and forms as used for the derivation set in the validation dataset. Instead we skipped this step and moved directly to the clinical application of a practicable and user-friendly form along with recommendations as to the consequences of the ratings to be considered. This design feature inhibits clearer delineation, whether the observed differences in the VAS performance were due to the different sample, differing professional experience amongst staff, the alteration of the design (scale versus ruler), the immediate feedback of the result on the score or the provided recommendations. A related problem is the lack of information on the factors considered by the nurses when rating the VAS. A second limitation is the small number of events that prevented the calculation of more elaborate statistical models accounting for other patient covariates such as diagnosis or demographic variables. We are currently addressing the first problem by means of a qualitative research project, in which nursing staff is interrogated about the thoughts and considerations leading to a specific subjective risk assessment. This project will reveal whether subjective risk assessment is actually incorporating actuarial data such as knowledge about prior patient behavior. Finally, providing an interpretation and suggestion for action with the score result partially violates the condition of independence between outcome and prediction. If only the occurrence of attacks is considered as an outcome event, cases of attacks prevented by interventions initiated as a consequence of the rating may inflate the false positive rate. In contrast, the composite outcome (attacks and interventions initiated following the rating) overestimates the true positive rate. It is reassuring that the area under the Receiver Operating Characteristic curve using either outcome definition differed only by a small margin (0.90 versus 0.86). It should also be noted that the performance of the VAS in the validation dataset was similar to that of earlier reports from other investigations [14].

In summary, we ascertained satisfactory performance of the BVC in an independent dataset where multilevel likelihood ratio based interpretations and action plans were provided. Adding a visual analogue scale for subjective risk assessment appeared to improve the compliance of the staff with systematic risk prediction but did not result in improved test accuracy in the validation dataset. The considerable difference in test performance for the visual analogue scale between the application within a research framework (derivation dataset) and the use in daily practice warrant further scrutiny. The combined instrument is currently been tested in a multi-center randomized controlled trial to assess the efficacy of systematic risk assessment. Until these data are available the recommendation for routine use cannot be extended from the BVC risk assessment to the combined BVC-VAS instrument. Finally, it should be born in mind that attacks are rare events. Even the use of the BVC-VAS may imply that about half of the attacks will not be properly predicted and that only about 1 in 10 of all patients classified as moderate or high risk would indeed have proceeded to commit an attack.

## Conclusion

The BVC-VAS is an easy to use and accurate instrument for systematic short-term prediction of violent attacks in acute psychiatric wards. The inclusion of the VAS-derived data did not change the accuracy of the original BVC. Further research is needed on the factors considered by the nurses when rating the VAS and on the preventive efficacy of using the BVC-VAS.

## Appendix

Preventive Measures to consider:

- No specific measures to prevent an attack

- Careful observation

- General conversation (directed to reduce aggression)

- Walk outdoors 1:1 (directed to reduce aggression)

- Walk outdoors in a group (directed to reduce aggression)

- Reduction of demands (e.g. participation in activities)

- Relaxation exercise

- Confrontation with ward rules

- Discussion of risk with patient

- Talk-down (to deescalate)

- Transfer to intensive area within ward

- 1:1-observation for several hours

- Increase of medication dosage

- PRN-medication per os (psychotropic drugs)

- Open isolation in the patients own room (time out)

- Preventive seclusion (closed seclusion room)

- Injection of psychotropic drugs (forced/voluntary)

- Physical restraint (indicate nr. of points)

## Competing interests

The author(s) declare that they have no competing interests.

## Authors' contributions

The authors have carried out the study together. CA was the principal investigator in this study and collected and organized the data. Together with IN and JF he wrote the first draft of the article. JF and IN contributed to the conception of the study and to the statistical analysis and interpretation of the data. TD, RH and HJH contributed to the conception of the study and revised the manuscript critically. All authors read and approved the manuscript.

## Pre-publication history

The pre-publication history for this paper can be accessed here:


